# Integrated left ventricular geometry–function phenotypes and long-term outcomes after acute myocardial infarction

**DOI:** 10.3389/fcvm.2026.1863946

**Published:** 2026-06-22

**Authors:** Seonmin Park, Seok Oh, Yongwhan Lim, Joon Ho Ahn, Dae Young Hyun, Seung Hun Lee, Kyung Hoon Cho, Min Chul Kim, Doo Sun Sim, Ju Han Kim, Youngkeun Ahn, Myung Ho Jeong, Jong Pil Park, Jay Young Rhew, Young Joon Hong

**Affiliations:** 1 Department of Cardiology, Presbyterian Medical Center, Jeonju, Republic of Korea; 2 Department of Cardiology, Chonnam National University Hospital, Gwangju, Republic of Korea; 3 Department of Cardiology, Chonnam National University Medical School, Gwangju, Republic of Korea; 4 Department of Cardiology, Gwangju Veterans Hospital, Gwangju, Republic of Korea

**Keywords:** acute myocardial infarction, echocardiography, left ventricular remodeling, outcomes, prognosis

## Abstract

**Background/aims:**

Left ventricular (LV) remodeling after acute myocardial infarction (AMI) is a key determinant of long-term outcomes. However, it remains unclear how LV geometry and systolic function interact to influence prognosis in real-world populations. We investigated the combined prognostic impact of LV dilatation and systolic dysfunction in patients with AMI.

**Methods:**

We analyzed 19,664 patients with AMI from nationwide Korean multicenter registries. LV geometry-function phenotypes were defined according to LV end-diastolic dimension (LVEDD ≥53 mm for dilatation) and LV ejection fraction (LVEF ≤50% for reduced systolic function), and categorized as non-dilated/preserved (group A), dilated/preserved (group B), non-dilated/reduced (group C), and dilated/reduced (group D). The primary endpoint was 3-year major adverse cardiac and cerebrovascular events (MACCE), defined as cardiac death, non-fatal myocardial infarction, unplanned revascularization, cerebrovascular accident, and cardiovascular readmission. Associations between baseline geometry-function phenotypes and outcomes were assessed using multivariable Cox proportional hazards models.

**Results:**

The incidence of MACCE increased stepwise from group A to D (13.2%, 13.5%, 16.8%, and 21.9%, respectively). In fully adjusted analyses, LV dilatation was not associated with increased risk in patients with preserved LVEF [group B vs. group A: hazard ratio (HR), 1.13; 95% confidence interval, 0.99–1.29]. In contrast, LV dilatation was associated with a higher risk when accompanied by reduced LVEF [group C: HR, 1.12 (1.01–1.24); group D: HR, 1.44 (1.29–1.60)].

**Conclusions:**

The prognostic significance of LV dilatation after AMI depends on underlying systolic function. While LV dilatation alone may reflect a compensatory process in preserved LVEF, its presence in reduced LVEF identifies a high-risk phenotype. An integrated assessment of LV geometry and function may provide complementary risk stratification beyond conventional LVEF-based assessment.

## Introduction

Acute myocardial infarction (AMI) remains a major cause of morbidity and mortality worldwide, despite significant advances in reperfusion strategies and pharmacologic therapies (1–3). Although timely restoration of coronary blood flow markedly improves survival, many patients experience adverse structural and functional alterations of the left ventricle during the post-infarction period (4). These maladaptive changes, primarily driven by the extent of myocardial injury, neurohormonal activation, and hemodynamic stress, play a critical role in the transition from AMI to chronic heart failure (5, 6).

Left ventricular (LV) remodeling represents a complex process of structural and geometric adaptation following myocardial injury (4, 7). It is characterized by progressive ventricular dilatation, increased sphericity, and impaired contractile performance, which collectively contribute to unfavorable long-term outcomes. Echocardiographic parameters—particularly LV end-diastolic and end-systolic dimensions or volumes—serve as key tools for quantifying the extent of remodeling and evaluating its prognostic relevance.

Despite its clinical importance, real-world data regarding the impact of LV remodeling on long-term outcomes after AMI remain limited. Therefore, in the present study, we aimed to investigate the prognostic implications of LV remodeling in patients with AMI.

## Methods

### Study participants and study scheme

All the clinical data were derived from two Korean nationwide multicenter AMI observational databases [the Korea Acute Myocardial Infarction Registry-National Institutes of Health [KAMIR-NIH] and the Korea Acute Myocardial Infarction Registry-V [KAMIR-V]] (8–10). The KAMIR-NIH database includes Korean patients with AMI from November 1, 2011, to December 31, 2015, and the KAMIR-V database includes those from January 1, 2016, to June 30, 2020. These two cohorts included 20 and 33 percutaneous coronary intervention (PCI)- and coronary artery bypass grafting (CABG)-capable healthcare facilities, respectively (8, 9). The cohort protocol was designed in accordance with the Helsinki Declaration 2013 and approved by the ethics committee or institutional review board (IRB) of each participating facility.

**Figure 1:**
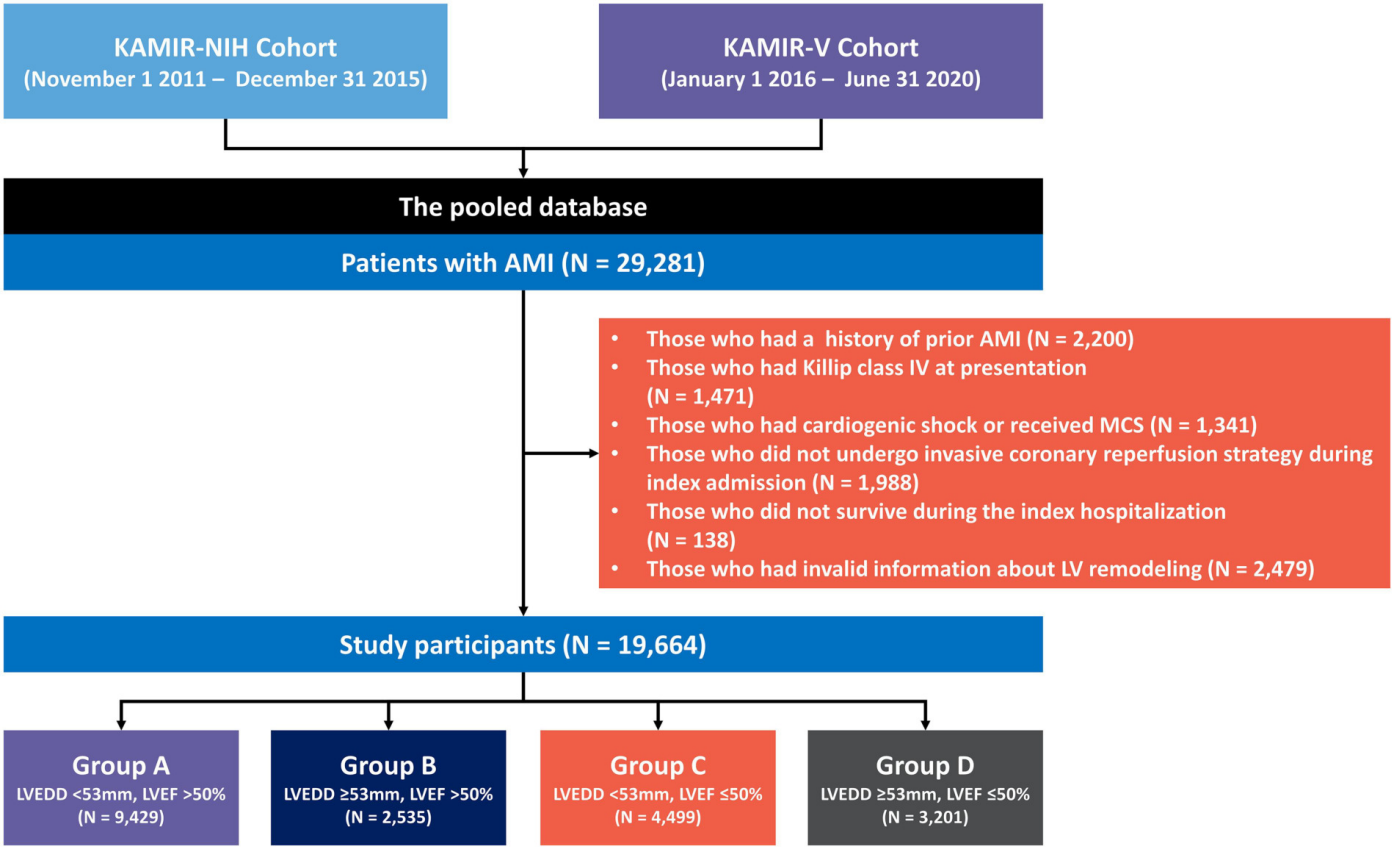
Study flow diagram. AMI, acute myocardial infarction; KAMIR-NIH, Korea acute myocardial infarction registry-national institutes of health; KAMIR-V, Korea acute myocardial infarction registry-V; LV, left ventricular; LVEDD, left ventricular end-diastolic dimension; LVEF, left ventricular ejection fraction; MCS, mechanical circulatory support.

A total of 29,281 Korean patients with AMI were screened. We excluded those who met the following criteria: a history of prior AMI (*n* = 2,200), Killip functional class IV at presentation (*n* = 1,471), cardiogenic shock or receipt of mechanical circulatory support (*n* = 1,341), no invasive coronary reperfusion strategy during the index admission (*n* = 1,988), in-hospital death (*n* = 138), and missing or implausible values required to define LV remodeling (*n* = 2,479). After applying these criteria, 19,664 patients were included in the final analytic cohort (Figure 1).

### Classification of LV geometry-function phenotypes

LV geometry-function phenotypes were defined by combining structural and functional echocardiographic parameters. Geometry was determined using the LV end-diastolic dimension (LVEDD); patients with LVEDD ≥ 53 mm were classified as dilated, whereas those with LVEDD < 53 mm were non-dilated. Function was assessed according to the LV ejection fraction (LVEF); LVEF ≤ 50% indicated reduced systolic function and LVEF > 50% indicated preserved function. These cutoff values were selected based on prior echocardiographic and clinical studies suggesting that LVEDD values in the range of approximately 52–55 mm may reflect early ventricular enlargement or adverse remodeling. In addition, the selected threshold lies near the upper-normal reference range proposed in current chamber quantification guidelines (11–13). Since indexed LV chamber dimensions were not consistently available in these cohort databases, an absolute LVEDD cutoff was used as a pragmatic operational criterion for phenotype classification in this large multicenter observational study.

Accordingly, four LV geometry-function phenotypes were established: (1) non-dilated/preserved (group A, LVEDD < 53 mm, LVEF > 50%), (2) dilated/preserved (group B, LVEDD ≥ 53 mm, LVEF > 50%), (3) non-dilated/reduced (group C, LVEDD < 53 mm, LVEF ≤ 50%), and (4) dilated/reduced (group D, LVEDD ≥ 53 mm, LVEF ≤ 50%) (Figure 1). These categories were used to examine the relationship between these patterns and clinical outcomes (Supplementary Data S1).

### Echocardiographic measurements and standardization

All echocardiographic parameters were measured according to standardized protocols recommended by the American Society of Echocardiography and the European Association of Cardiovascular Imaging (14, 15). Two-dimensional transthoracic echocardiography was performed during the index hospitalization using commercially available ultrasound systems at each participating institution.

The LVEF was calculated from apical two- and four-chamber views using the modified Simpson's biplane method and was cross-verified by visual estimation when image quality was suboptimal. The LVEDD and LV end-systolic dimension (LVESD) were obtained from the parasternal long-axis view at the level of the mitral valve leaflet tips using M-mode or two-dimensional imaging. All measurements were performed by experienced sonographers or cardiologists blinded to clinical outcomes.

### Definitions of baseline characteristics

Baseline characteristics were defined as previously reported (8). AMI was defined as myocardial damage evidenced by elevation in myocardial biomarkers accompanied by the following features: clinical symptoms or signs suggestive of myocardial ischemia; new-onset loss of myocardial viability or abnormal findings in regional wall motion confirmed via cardiovascular imaging; and evidence of intracoronary thrombus identified primarily by conventional coronary angiography according to operator interpretation. Intracoronary imaging modalities such as intravascular ultrasound or optical coherence tomography were not routinely mandated across participating centers. ST-segment elevation myocardial infarction (STEMI) was defined as AMI with new-onset ST-segment elevation in ≥ 2 continuous leads (> 0.2 mV in precordial leads V1–V3 or > 0.1 mV in all the other leads of the 12-lead electrocardiogram).

Smoking history, identified using a questionnaire, was defined as current or previous smoking. Estimated glomerular filtration rate was calculated using the 2021 CKD-EPI (Chronic Kidney Disease Epidemiology Collaboration) creatinine equation (16) based on the serum creatinine level at the time of the hospital visit. Comorbidities of interest included hypertension, diabetes mellitus, dyslipidemia, prior coronary artery disease (CAD) (not AMI), prior heart failure, and prior cerebrovascular accident (CVA).

The angiographic and procedural characteristics of the participants were retrospectively collected. The infarct-related artery was defined as the epicardial coronary artery liable for the clinical manifestation of AMI after atherothrombotic occlusion and/or stenosis. While lesion characteristics of the infarct-related artery were stratified based on the American College of Cardiology/the American Heart Association (ACC/AHA) classification (17), the degree of intracoronary flow of the infarct-related artery was stratified based on the Thrombolysis In Myocardial Infarction flow grading system (18). Significant stenosis of the epicardial coronary artery was defined as reductions of ≥ 50% and ≥ 70% in the arterial lumen diameter of the left main coronary artery (LMCA) and other epicardial coronary arteries. Multivessel CAD was defined as the presence of ≥ 2 significant stenoses of the epicardial coronary arteries (≥ 70% stenosis of ≥ 2 epicardial coronary arteries or ≥ 50% stenosis of the LMCA with ≥ 1 epicardial coronary artery). Treatment strategies of reperfusion were categorized into three types: stenting (bare-metal stent, drug-eluting stent, and/or bioresorbable vascular scaffold); balloon angioplasty alone; and other invasive coronary reperfusion procedures without stent implantation or balloon angioplasty, such as thrombus aspiration or adjunctive intracoronary pharmacologic therapy.

Post-discharge medications included aspirin, P2Y12 inhibitors, beta-blockers, renin-angiotensin-aldosterone system inhibitors, and statins.

### Study outcomes and follow-up

The primary endpoint was major adverse cardiac and cerebrovascular event (MACCE). MACCE was defined as a composite of cardiac death, non-fatal myocardial infarction (NFMI), any unplanned revascularization, CVA, and cardiovascular readmission. All-cause death was defined as a composite of both cardiac and non-cardiac deaths. Any unplanned revascularization was defined as either PCI for any coronary segment or CABG. Cardiovascular readmission was defined as any unplanned hospitalization due to worsening heart failure, recurrent ischemic symptoms, or other cardiac causes requiring inpatient care. Secondary endpoints included each individual component of MACCE and other components.

The clinical follow-up duration for each patient was approximately 36 months. It was conducted at 6 months, 1 year, 2 years, and 3 years via outpatient visits or whenever any cardiovascular event occurred (8, 19). Follow-up was censored on the date of the study outcome, date of death, or the end of the study period.

### Statistical analyses

Statistical analyses were performed to evaluate differences in baseline characteristics and long-term clinical outcomes among the four LV geometry-function phenotypes (Groups A–D). All analyses were conducted using SPSS (version 25.0; IBM Corp., Armonk, NY, USA) and Stata (version 15.0; StataCorp, College Station, TX, USA).

Continuous variables are presented as mean ± standard deviation for normally distributed data or median with interquartile range for non-normally distributed data. Categorical variables are expressed as frequencies with percentages. Inter-group comparisons were made using one-way analysis of variance or the Kruskal–Wallis test for continuous variables and the chi-square test for categorical variables. A two-sided *P* value < 0.05 was considered statistically significant.

Time-to-event outcomes were analyzed using Cox proportional hazards regression to estimate hazard ratios (HRs) and 95% confidence intervals (CIs) for each endpoint according to LV geometry-function phenotype. Three models were sequentially fitted: (1) an unadjusted model, (2) an age- and sex-adjusted model, and (3) a fully adjusted multivariable model including demographic, clinical, angiographic, procedural, and pharmacologic covariates potentially influencing outcomes.

Details of all covariates included in the fully adjusted model are summarized in Supplementary Data S2. In brief, variables reflecting LV geometry or systolic function (LVEDD, LVEF, and LVESD) were not simultaneously included in the multivariable model because LVEDD and LVEF jointly defined the exposure phenotype (geometry   ×   function). Given the close mathematical and physiological relationship between LVESD, LVEDD, and LVEF, additional adjustment for LVESD could introduce construct redundancy and collinearity. Therefore, LVESD was only presented descriptively in Table 1 for clinical context but was not included in the fully adjusted model to avoid potential over-adjustment.

**Table 1:** Baseline characteristics of study participants according to left ventricular geometry–function phenotypes.

Variable	Group A	Group B	Group C	Group D	*P*-value
	(*n* = 9,429)	(*n* = 2,535)	(*n* = 4,499)	(*n* = 3,201)	for trend
Age, years	62.59 ± 12.18	60.63 ± 12.01	64.76 ± 12.72	64.31 ± 12.59	<0.001
Age ≥75 years	1,806 (19.2)	385 (15.2)	1,204 (26.8)	783 (24.5)	<0.001
Male sex	7,112 (75.4)	2,246 (88.6)	3,173 (70.5)	2,587 (80.8)	0.133
Use of EMS	1,541 (16.3)	422 (16.7)	812 (18.1)	486 (15.2)	0.985
BMI, kg/m^2^	24.26 ± 3.20	25.49 ± 3.41	23.59 ± 3.27	24.45 ± 3.44	<0.001
Killip class II-III	903 (9.7)	255 (10.3)	777 (17.5)	770 (24.5)	<0.001
Comorbidities					
Hypertension	4,522 (48.0)	1,237 (48.8)	2,093 (46.5)	1,632 (51.0)	0.125
Diabetes mellitus	2,329 (24.7)	607 (23.9)	1,159 (25.8)	984 (30.7)	<0.001
Dyslipidemia	1,280 (13.6)	328 (12.9)	463 (10.3)	392 (12.3)	<0.001
Prior CAD	725 (7.7)	190 (7.5)	282 (6.3)	260 (8.1)	0.453
Prior heart failure	61 (0.7)	18 (0.7)	37 (0.8)	65 (2.0)	<0.001
Prior CVA	517 (5.5)	129 (5.1)	299 (6.7)	221 (6.9)	<0.001
Smoking history	5,280 (57.5)	1,697 (68.5)	2,389 (54.7)	1,883 (60.3)	0.659
Family history of CAD	781 (8.5)	202 (8.2)	285 (6.5)	196 (6.3)	<0.001
Use of thrombolysis	51 (0.5)	20 (0.8)	39 (0.9)	49 (1.5)	<0.001
Multivessel CAD	4,627 (49.1)	1,230 (48.5)	2,293 (51.0)	1,891 (59.1)	<0.001
LMCA disease	377 (4.0)	93 (3.7)	180 (4.0)	155 (4.8)	0.103
Femoral approach	4,447 (47.2)	1,065 (42.0)	2,538 (56.4)	1,609 (50.3)	<0.001
Use of GPIIb/IIIa inhibitors	901 (9.6)	346 (13.7)	514 (11.4)	461 (14.4)	<0.001
Use of thrombus aspiration	1,533 (16.3)	428 (16.9)	1,055 (23.5)	546 (17.1)	<0.001
Use of intracoronary imaging	2,301 (24.4)	776 (30.6)	1,000 (22.2)	863 (27.0)	0.435
Infarct-related artery					<0.001
LMCA	180 (1.9)	51 (2.0)	84 (1.9)	84 (2.6)	
LAD	3,876 (41.1)	950 (37.5)	2,787 (61.9)	1,807 (56.5)	
LCX	1,899 (20.1)	609 (24.0)	543 (12.1)	513 (16.0)	
RCA	3,474 (36.8)	925 (36.5)	1,085 (24.1)	797 (24.9)	
ACC/AHA lesion type B2/C	7,703 (84.5)	2,118 (85.9)	3,719 (86.4)	2,709 (87.7)	<0.001
PCI strategies					<0.001
Stenting	8,778 (94.2)	2,379 (94.8)	4,282 (96.0)	3,017 (95.2)	
Balloon angioplasty alone	479 (5.1)	110 (4.4)	151 (3.4)	130 (4.1)	
Others	59 (0.6)	21 (0.8)	29 (0.6)	23 (0.7)	
eGFR, mL/min/1.73m^2^	83.77 ± 22.41	84.68 ± 23.11	79.59 ± 23.52	74.57 ± 26.81	<0.001
Echocardiographic profiles					
LVEF, %	59.91 ± 6.07	58.30 ± 5.73	43.87 ± 5.56	40.76 ± 7.63	<0.001
LVESD, mm	30.02 ± 5.52	36.37 ± 6.15	33.04 ± 7.83	42.78 ± 9.23	<0.001
LVEDD, mm	46.85 ± 4.77	55.73 ± 13.67	47.15 ± 6.08	57.40 ± 10.27	<0.001
STEMI as a final diagnosis	3,902 (41.4)	1,054 (41.6)	2,891 (64.3)	1,747 (54.6)	<0.001
Medications					
Aspirin	9,412 (99.8)	2,530 (99.8)	4,493 (99.9)	3,198 (99.9)	0.258
P2Y12 inhibitors	9,407 (99.8)	2,527 (99.7)	4,487 (99.7)	3,193 (99.8)	0.784
Beta-blockers	7,884 (83.6)	2,033 (80.2)	3,833 (85.2)	2,664 (83.2)	0.426
RAAS inhibitors	8,854 (93.9)	2,375 (93.7)	4,165 (92.6)	3,001 (93.8)	0.110
Statins	9,154 (97.1)	2,445 (96.5)	4,307 (95.7)	3,064 (95.7)	<0.001

Continuous variables are presented as mean ± standard deviation and categorical variables as numbers with percentages. Group definitions were based on combined geometric and functional classification using alternative thresholds to assess the robustness of the primary analysis. ACC/AHA, American college of cardiology/American heart association; BMI, body mass index; CAD, coronary artery disease; CVA, cerebrovascular accident; eGFR, estimated glomerular filtration rate; EMS, emergency medical service; GPIIb/IIIa, glycoprotein IIb/IIIa; LAD, left anterior descending coronary artery; LCX, left circumflex coronary artery; LMCA, left main coronary artery; LVEDD, left ventricular end-diastolic diameter; LVEF, left ventricular ejection fraction; LVESD, left ventricular end-systolic diameter; PCI, percutaneous coronary intervention; RAAS, renin-angiotensin-aldosterone system; RCA, right coronary artery; STEMI, ST-segment elevation myocardial infarction.

Model assumptions of proportional hazards were verified using Schoenfeld residuals and log-minus-log survival plots.

Restricted cubic spline functions were incorporated into multivariable Cox proportional hazards models to evaluate potential non-linear associations between LVEDD, LVEF, and the risk of MACCE. Three knots were placed at the 10th, 50th, and 90th percentiles of the variable distributions. Overall and non-linear associations were assessed using Wald tests.

Kaplan–Meier survival curves were constructed to depict cumulative incidence of events and were compared among groups using the log-rank test. Patients with missing covariate data or without post-discharge follow-up were excluded from the multivariable analyses.

### Ethics statement

The present study was conducted according to the ethical standards of the World Medical Association's Declaration of Helsinki. This study was ratified by the IRB of Chonnam National University Hospital (IRB No. CNUH-2025-020). Since this study was a *post-hoc* retrospective analysis of de-identified data (the KAMIR-NIH and KAMIR-V cohorts), the IRB waived the need for informed consent.

## Results

### Baseline patient characteristics

A total of 19,664 patients with AMI were included in the final analytic cohort (Figure 1). Patients were categorized into four groups according to LV geometry and systolic function: group A (non-dilated & preserved; *n* = 9,429), B (dilated & preserved; *n* = 2,535), C (non-dilated & reduced; *n* = 4,499), and D (dilated & reduced; *n* = 3,201). Baseline characteristics of the participants are summarized in Table 1.

Patients in groups B and D tended to be older and exhibited a higher prevalence of hypertension and diabetes mellitus, whereas those in group C comprised a greater proportion of men and current smokers. The frequency of multivessel CAD and higher Killip functional class increased progressively from group A to D, paralleling the stepwise deterioration in ventricular geometry and systolic performance. In addition, patients with reduced LVEF (groups C and D) showed a higher prevalence of STEMI and left anterior descending artery involvement compared with those with preserved LVEF (groups A and B). Echocardiographic indices showed graded increases in LV dimensions and corresponding reductions in LVEF across the groups, consistent with the predefined patterns. Prescription rates of evidence-based medical therapy were uniformly high among all groups.

### Study outcomes

Clinical outcomes according to LV geometry-function phenotypes are summarized in Table 2. The cumulative incidence of MACCE demonstrated a graded increase across the four groups, ranging from 13.2% in group A to 21.9% in group D. In the fully adjusted model, the risk of MACCE did not differ significantly between groups A and B (HR, 1.13; 95% CI, 0.99–1.29). In contrast, patients with reduced LVEF showed higher event rates, with groups C (HR, 1.12; 95% CI, 1.01–1.24) and D (HR, 1.44; 95% CI, 1.29–1.60) exhibiting progressively increasing risks compared with group A.

**Table 2:** HRs (95% CI) for the associations between groups and incidences of clinical outcomes.

Outcome			HR with 95% CI		
	Study group	Events, *n* (%)	Unadjusted model	Age-sex adjusted model	Fully-adjusted model
MACCE	Group A	1,224 (13.2)	1 (reference)	1 (reference)	1 (reference)
	Group B	337 (13.5)	1.02 (0.90–1.15)	1.11 (0.98–1.25)	1.13 (0.99–1.29)
	Group C	731 (16.8)	1.32 (1.20–1.44)	1.22 (1.12–1.34)	1.12 (1.01–1.24)
	Group D	678 (21.9)	1.78 (1.62–1.95)	1.71 (1.56–1.88)	1.44 (1.29–1.60)
All-cause death	Group A	344 (3.7)	1 (reference)	1 (reference)	1 (reference)
	Group B	86 (3.5)	0.92 (0.73–1.17)	1.07 (0.84–1.35)	1.16 (0.90–1.50)
	Group C	267 (6.1)	1.70 (1.45–1.99)	1.43 (1.22–1.68)	1.29 (1.08–1.55)
	Group D	272 (8.8)	2.46 (2.10–2.88)	2.17 (1.85–2.54)	1.63 (1.35–1.96)
Cardiac death	Group A	157 (1.7)	1 (reference)	1 (reference)	1 (reference)
	Group B	31 (1.2)	0.73 (0.50–1.07)	0.84 (0.57–1.24)	0.90 (0.59–1.37)
	Group C	135 (3.1)	1.88 (1.49–2.36)	1.58 (1.26–2.00)	1.34 (1.03–1.73)
	Group D	149 (4.8)	2.94 (2.35–3.69)	2.60 (2.07–3.25)	1.87 (1.44–2.42)
Non-cardiac death	Group A	187 (2.0)	1 (reference)	1 (reference)	1 (reference)
	Group B	55 (2.2)	1.09 (0.80–1.47)	1.26 (0.93–1.70)	1.37 (0.99–1.90)
	Group C	132 (3.0)	1.55 (1.24–1.93)	1.30 (1.04–1.63)	1.25 (0.97–1.61)
	Group D	123 (4.0)	2.05 (1.63–2.57)	1.80 (1.43–2.27)	1.41 (1.08–1.84)
NFMI	Group A	215 (2.3)	1 (reference)	1 (reference)	1 (reference)
	Group B	56 (2.2)	0.96 (0.72–1.29)	1.02 (0.76–1.37)	0.92 (0.66–1.29)
	Group C	106 (2.4)	1.08 (0.85–1.36)	1.03 (0.81–1.30)	0.96 (0.74–1.25)
	Group D	91 (2.9)	1.32 (1.03–1.69)	1.29 (1.01–1.65)	1.03 (0.78–1.36)
Any unplanned revascularization	Group A	649 (7.0)	1 (reference)	1 (reference)	1 (reference)
	Group B	183 (7.3)	1.04 (0.88–1.23)	1.04 (0.88–1.23)	1.04 (0.87–1.24)
	Group C	288 (6.6)	0.97 (0.84–1.11)	0.97 (0.84–1.11)	0.95 (0.81–1.11)
	Group D	243 (7.9)	1.17 (1.01–1.36)	1.17 (1.01–1.36)	1.08 (0.92–1.27)
CVA	Group A	148 (1.6)	1 (reference)	1 (reference)	1 (reference)
	Group B	47 (1.9)	1.17 (0.84–1.63)	1.32 (0.95–1.83)	1.43 (1.00–2.05)
	Group C	80 (1.8)	1.18 (0.90–1.55)	1.08 (0.82–1.42)	0.95 (0.69–1.30)
	Group D	68 (2.2)	1.43 (1.07–1.91)	1.39 (1.04–1.86)	1.25 (0.90–1.74)
Cardiovascular readmission	Group A	171 (1.8)	1 (reference)	1 (reference)	1 (reference)
	Group B	48 (1.9)	1.04 (0.75–1.43)	1.25 (0.91–1.73)	1.28 (0.89–1.82)
	Group C	171 (3.9)	2.20 (1.78–2.72)	1.91 (1.54–2.36)	1.62 (1.27–2.06)
	Group D	212 (6.8)	3.92 (3.21–4.80)	3.74 (3.05–4.58)	2.96 (2.34–3.73)

CI, confidence interval; CVA, cerebrovascular accident; HR, hazard ratio; MACCE, major adverse cardiac and cerebrovascular event; NFMI, non-fatal myocardial infarction.

A similar trend was observed for secondary outcomes. The incidence of all-cause death and cardiac death increased stepwise from groups A to D, with the highest risks consistently noted in group D. Differences in the incidences of NFMI, unplanned revascularization, and CVA were less pronounced, although groups with reduced LVEF generally demonstrated higher event rates.

**Figure 2:**
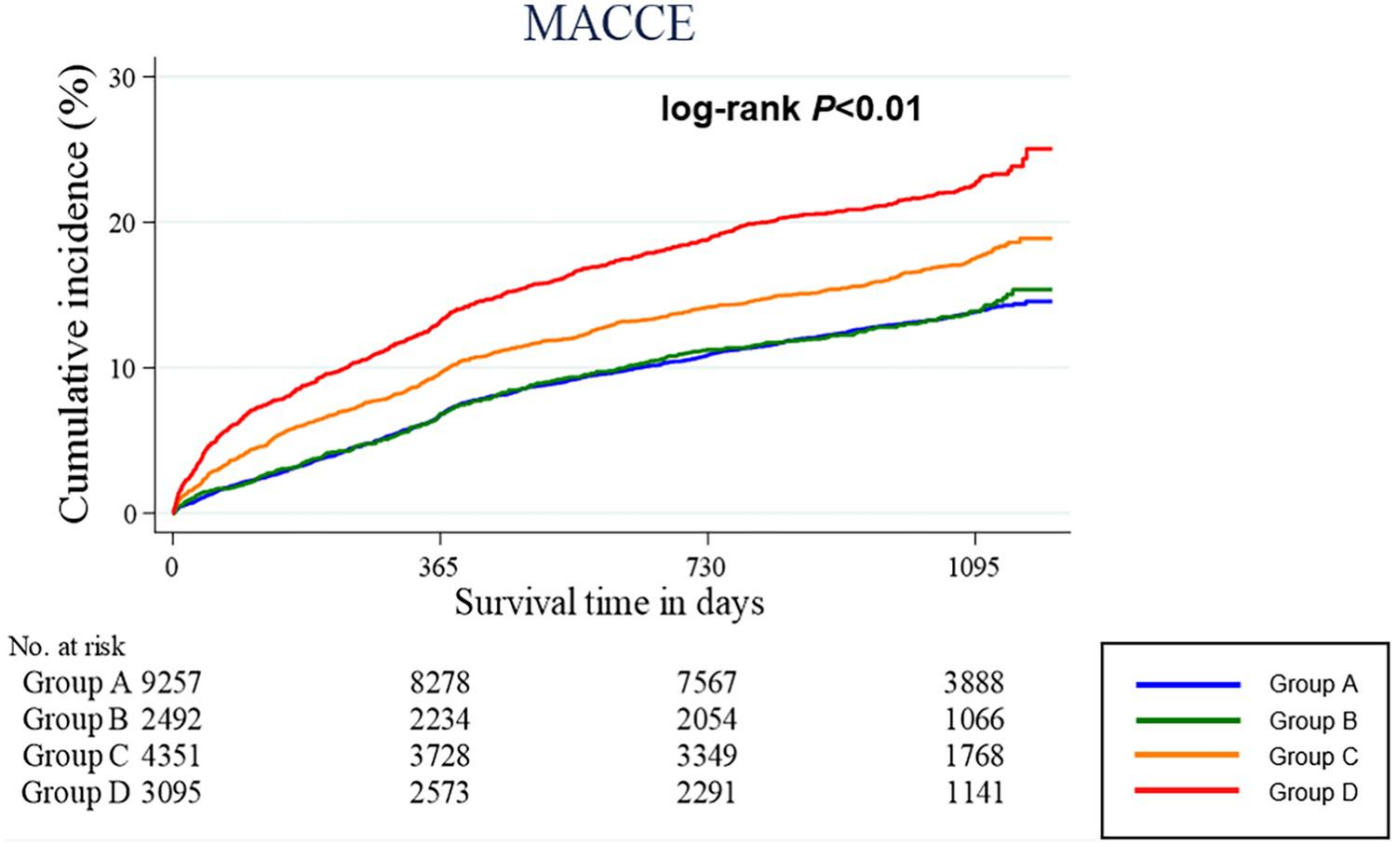
Kaplan–meier curves for MACCE according to LV geometry-function phenotypes. Blue line indicates group A (non-dilated/preserved), green line indicates group B (dilated/preserved), orange line indicates group C (non-dilated/reduced), and red line indicates group D (dilated/reduced). LV, left ventricular; MACCE, major adverse cardiac and cerebrovascular accident.

Kaplan–Meier survival curves illustrated clear separation between preserved- and reduced-function groups, particularly when LV dilatation was concomitantly present (Figure 2, Supplementary Figure S1).

### Restricted cubic spline analysis of LVEDD and MACCE

**Figure 3:**
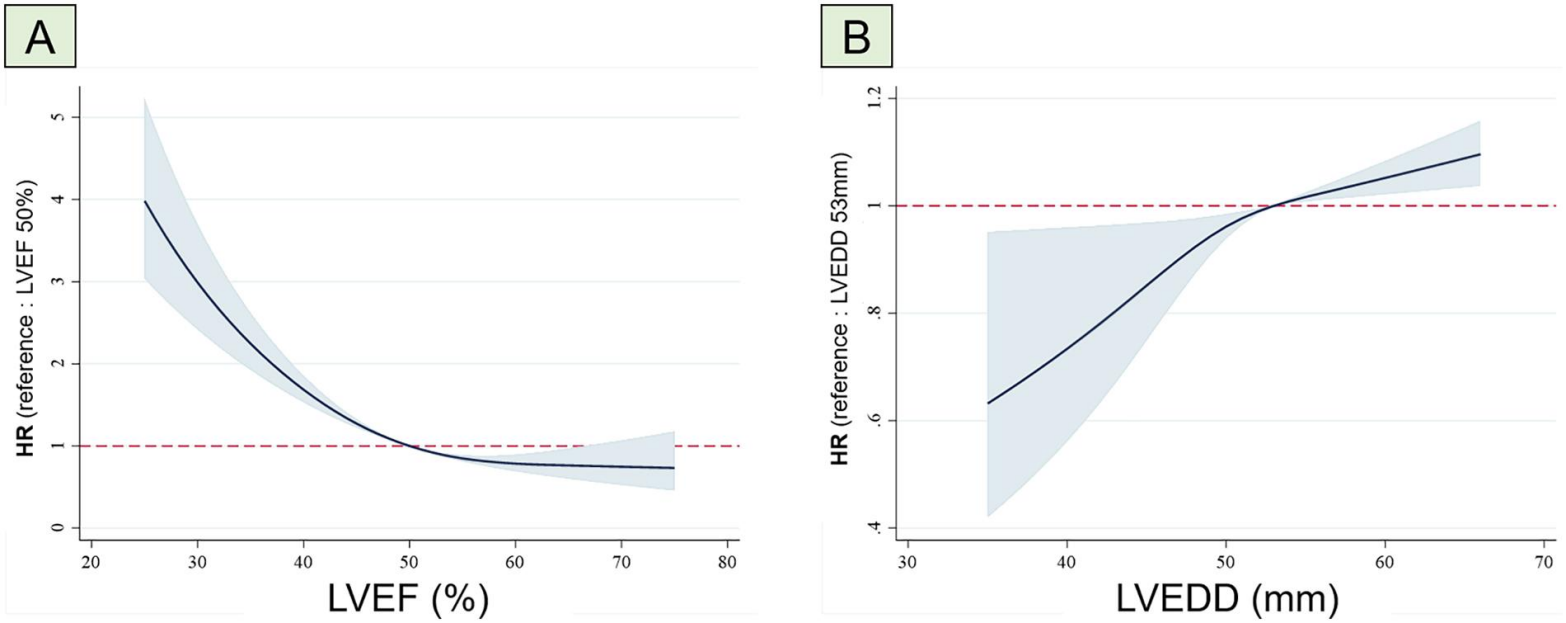
Restricted cubic spline analysis of the associations of LVEF and LVEDD with MACCE. **(A)** Adjusted hazard ratio for MACCE as a continuous function of LVEF (reference, LVEF 50%); risk increases as LVEF decreases. **(B)** Adjusted hazard ratio for MACCE as a continuous function of LVEDD (reference, LVEDD 53 mm); risk increases as LVEDD increases. Solid lines indicate point estimates and shaded areas the 95% confidence intervals. HR, hazard ratio; LVEDD, left ventricular end-diastolic dimension; LVEF, left ventricular ejection fraction; MACCE, major adverse cardiac and cerebrovascular events.

To evaluate the potential non-linear association between LVEF, LVEDD, and MACCE, a restricted cubic spline analysis was performed within a multivariable Cox proportional hazards model. In the restricted cubic spline Cox model, both LVEF and LVEDD were significantly associated with the risk of MACCE (overall association, *P* < 0.001), with evidence of a significant non-linear relationship (*P* for non-linearity < 0.001) (Figure 3).

### Sensitivity analysis using both alternative cutoffs of LVEF and LVEDD

In the sensitivity analyses using alternative cutoffs for LVEDD and LVEF (LVEDD ≥55 mm and LVEF <40%), the overall direction and magnitude of the associations remained broadly consistent with those of the primary analysis (Supplementary Table S1, S2).

### Subgroup-Specific analysis of MACCE

Subgroup analyses for MACCE are presented in Table 3. Across most subgroups, the relative risks associated with each LV geometry-function phenotype were generally consistent with the overall results, with groups characterized by reduced systolic function (groups C and D) showing higher event rates than those with preserved function (groups A and B).

**Table 3:** Exploratory subgroup analysis comparing HRs for MACCE according to each group (fully-adjusted model).

Subgroup	Study group (LVEF category)	Events, *n* (%)	HR (95% CI)	*P*-value	Interaction *P*-value
Age					0.956
≥75 years	Group A	396 (22.5)	1 (reference)		
	Group B	82 (22.2)	1.12 (0.86–1.45)	0.397	
	Group C	309 (27.0)	1.16 (0.98–1.37)	0.090	
	Group D	246 (33.4)	1.45 (1.20–1.74)	<0.001	
<75 years	Group A	828 (11.0)	1 (reference)		
	Group B	255 (12.0)	1.14 (0.98–1.33)	0.088	
	Group C	422 (13.2)	1.11 (0.98–1.27)	0.106	
	Group D	432 (18.3)	1.44 (1.26–1.64)	<0.001	
Sex					0.043
Male	Group A	844 (12.1)	1 (reference)		
	Group B	283 (12.8)	1.07 (0.93–1.24)	0.355	
	Group C	424 (13.8)	0.99 (0.87–1.13)	0.933	
	Group D	506 (20.2)	1.39 (1.23–1.58)	<0.001	
Female	Group A	380 (16.7)	1 (reference)		
	Group B	54 (18.9)	1.32 (0.98–1.78)	0.070	
	Group C	307 (23.9)	1.37 (1.16–1.63)	<0.001	
	Group D	172 (29.1)	1.55 (1.26–1.90)	<0.001	
BMI					0.856
≥25 kg/m^2^	Group A	378 (11.4)	1 (reference)		
	Group B	142 (11.6)	1.06 (0.86–1.30)	0.585	
	Group C	159 (13.0)	1.07 (0.87–1.31)	0.540	
	Group D	209 (17.6)	1.35 (1.12–1.63)	0.002	
<25 kg/m^2^	Group A	782 (14.2)	1 (reference)		
	Group B	183 (15.7)	1.14 (0.96–1.35)	0.135	
	Group C	526 (18.2)	1.15 (1.02–1.30)	0.023	
	Group D	446 (25.4)	1.48 (1.30–1.68)	<0.001	
Killip class					0.246
II -III	Group A	170 (19.2)	1 (reference)		
	Group B	47 (19.3)	1.21 (0.85–1.71)	0.284	
	Group C	196 (26.0)	1.23 (0.98–1.55)	0.076	
	Group D	259 (34.8)	1.64 (1.31–2.03)	<0.001	
I	Group A	1,043 (12.6)	1 (reference)		
	Group B	285 (12.9)	1.11 (0.97–1.28)	0.141	
	Group C	529 (14.9)	1.11 (0.99–1.25)	0.072	
	Group D	411 (17.8)	1.35 (1.19–1.53)	<0.001	
Hypertension					0.147
Yes	Group A	707 (15.9)	1 (reference)		
	Group B	217 (17.8)	1.28 (1.08–1.51)	0.004	
	Group C	412 (20.4)	1.11 (0.97–1.27)	0.145	
	Group D	434 (27.7)	1.49 (1.30–1.71)	<0.001	
No	Group A	517 (10.7)	1 (reference)		
	Group B	120 (9.4)	0.95 (0.77–1.18)	0.648	
	Group C	319 (13.7)	1.13 (0.96–1.32)	0.131	
	Group D	244 (16.0)	1.34 (1.13–1.59)	0.001	
Diabetes mellitus					0.794
Yes	Group A	411 (18.0)	1 (reference)		
	Group B	113 (18.9)	1.08 (0.86–1.36)	0.494	
	Group C	245 (22.0)	1.06 (0.88–1.26)	0.555	
	Group D	287 (30.5)	1.42 (1.19–1.69)	<0.001	
No	Group A	813 (11.6)	1 (reference)		
	Group B	224 (11.8)	1.16 (0.99–1.36)	0.067	
	Group C	486 (15.0)	1.16 (1.02–1.32)	0.022	
	Group D	391 (18.1)	1.46 (1.27–1.67)	<0.001	
Prior CAD					0.964
Yes	Group A	145 (20.4)	1 (reference)		
	Group B	48 (25.8)	1.20 (0.83–1.73)	0.335	
	Group C	76 (28.5)	1.23 (0.90–1.68)	0.198	
	Group D	87 (34.7)	1.46 (1.07–2.00)	0.018	
No	Group A	1,079 (12.6)	1 (reference)		
	Group B	289 (12.5)	1.12 (0.97–1.29)	0.122	
	Group C	655 (16.0)	1.11 (0.99–1.24)	0.066	
	Group D	591 (20.8)	1.42 (1.27–1.59)	<0.001	
Prior heart failure					0.945
Yes	Group A	24 (41.4)	1 (reference)		
	Group B	7 (43.7)	1.77 (0.62–5.04)	0.282	
	Group C	19 (51.3)	0.97 (0.42–2.26)	0.946	
	Group D	30 (49.2)	2.19 (1.05–4.59)	0.038	
No	Group A	1,197 (13.0)	1 (reference)		
	Group B	329 (13.3)	1.13 (0.99–1.29)	0.069	
	Group C	712 (16.5)	1.11 (1.00–1.23)	0.050	
	Group D	644 (21.3)	1.43 (1.28–1.59)	<0.001	
Prior CVA					0.960
Yes	Group A	107 (21.2)	1 (reference)		
	Group B	28 (22.4)	1.33 (0.85–2.07)	0.210	
	Group C	79 (27.3)	1.19 (0.86–1.65)	0.304	
	Group D	78 (36.6)	1.60 (1.14–2.25)	0.007	
No	Group A	1,110 (12.7)	1 (reference)		
	Group B	306 (13.0)	1.12 (0.97–1.28)	0.118	
	Group C	651 (16.1)	1.11 (1.00–1.24)	0.054	
	Group D	597 (20.8)	1.43 (1.27–1.60)	<0.001	
Multivessel CAD					0.774
Yes	Group A	746 (16.4)	1 (reference)		
	Group B	205 (17.0)	1.14 (0.96–1.35)	0.129	
	Group C	465 (20.9)	1.15 (1.01–1.31)	0.036	
	Group D	490 (26.9)	1.51 (1.33–1.72)	<0.001	
No	Group A	478 (10.1)	1 (reference)		
	Group B	132 (10.3)	1.14 (0.93–1.41)	0.215	
	Group C	266 (12.5)	1.09 (0.92–1.30)	0.297	
	Group D	188 (14.8)	1.32 (1.10–1.60)	0.003	
eGFR					0.715
<60 mL/min/1.73 m^2^	Group A	326 (25.7)	1 (reference)		
	Group B	84 (28.3)	1.26 (0.97–1.64)	0.080	
	Group C	242 (29.4)	1.15 (0.95–1.38)	0.149	
	Group D	298 (37.8)	1.55 (1.30–1.87)	<0.001	
≥60 mL/min/1.73 m^2^	Group A	894 (11.2)	1 (reference)		
	Group B	250 (11.4)	1.11 (0.95–1.29)	0.197	
	Group C	489 (13.9)	1.12 (0.99–1.27)	0.070	
	Group D	380 (16.5)	1.40 (1.23–1.60)	<0.001	
Final diagnosis					0.736
STEMI	Group A	484 (12.7)	1 (reference)		
	Group B	117 (11.3)	1.05 (0.85–1.30)	0.654	
	Group C	430 (15.4)	1.03 (0.89–1.20)	0.676	
	Group D	310 (18.3)	1.35 (1.14–1.58)	<0.001	
NSTEMI	Group A	740 (13.6)	1 (reference)		
	Group B	220 (15.1)	1.19 (1.00–1.40)	0.044	
	Group C	301 (19.3)	1.19 (1.02–1.38)	0.022	
	Group D	368 (26.2)	1.47 (1.27–1.69)	<0.001	

BMI, body mass index; CAD, coronary artery disease; CI, confidence interval; CVA, cerebrovascular accident; eGFR, estimated glomerular filtration rate; HR, hazard ratio; LVEF, left ventricular ejection fraction; MACCE, major adverse cardiac and cerebrovascular accident; NSTEMI, non-ST-segment elevation myocardial infarction; STEMI, ST-segment elevation myocardial infarction.

A significant interaction was observed for sex (*P* for interaction = 0.043). In sex-stratified analyses, women demonstrated a clearer risk gradient across these phenotypes, with both groups C and D showing higher adjusted risks of MACCE compared with group A. In men, the risk increase was primarily limited to group D, whereas group C had risk estimates comparable to those of group A.

No other significant interactions were identified across the remaining subgroups, and the direction and magnitude of associations between the geometry-function phenotypes and MACCE were generally similar across all demographic and clinical categories.

## Discussion

In this large, nationwide cohort study of patients with AMI, the prognostic impact of structural left ventricular dilatation was dependent on the underlying systolic function. Among patients with preserved LVEF (> 50%; groups A and B), LV dilatation (LVEDD ≥ 53 mm) was not independently associated with a higher risk of MACCE or mortality. In contrast, among those with reduced LVEF (≤ 50%; groups C and D), concomitant LV dilatation was associated with a stepwise increase in adverse outcomes, with group D demonstrating the highest risk. These findings suggest that concurrent ventricular dilatation and impaired contractile function constitute a distinctly high-risk phenotype.

In the present study, we selected an LVEDD cutoff value of 53 mm to define ventricular dilatation when classifying LV geometry–function phenotypes. This cutoff was based on previously reported echocardiographic ranges and clinical literature (11–13, 20–22). In several studies, LVEDD values of approximately 52–55 mm have been used to indicate ventricular enlargement or early structural remodeling (13, 20–23). Therefore, 53 mm was considered a pragmatic threshold, allowing operational classification of LV geometry in a large observational dataset. Importantly, restricted cubic spline analysis confirmed a significant non-linear association between LVEDD and risk of MACCE (overall *P* < 0.001; *P* for non-linearity < 0.001), suggesting that LVEDD reflects a continuous risk spectrum rather than a strict dichotomous boundary, supporting the use of 53 mm as a cutoff value for phenotype classification.

In the sensitivity analyses using more stringent cutoffs (LVEF < 40% and LVEDD ≥ 55 mm), LV dilatation was associated with a significantly higher risk of adverse outcomes even among patients with preserved systolic function (group B), whereas no such association was observed with conventional thresholds. This finding suggests that the prognostic impact of LV dilatation depends on its severity, with mild dilatation potentially representing a less advanced stage of ventricular remodeling and more advanced dilatation indicating maladaptive remodeling. Accordingly, LV geometry appears to confer risk along a continuum rather than as a binary condition.

Several baseline characteristics exhibited a progressive gradient across the baseline geometry-function phenotypes. Patients with reduced systolic function—particularly those in group D—were older, had a higher prevalence of diabetes mellitus and prior heart failure, and more frequently presented with higher Killip functional class. In addition, multivessel CAD, ACC/AHA type B2/C lesions, and impaired renal function were more common in the dilated and reduced-LVEF groups (groups C and D), suggesting that advanced geometry-function phenotypes reflect not only intrinsic ventricular structural alterations but also a greater systemic atherosclerotic burden and hemodynamic compromise. The relatively lower use of beta-blockers in group D may also reflect poorer hemodynamic tolerance or clinician reluctance to initiate intensive neurohormonal blockade during the acute phase of AMI in patients with advanced ventricular dysfunction or heart failure. The high involvement of the left anterior descending coronary artery as the infarct-related artery, as well as the greater frequency of STEMI among reduced-LVEF groups, further supports the presence of more extensive myocardial involvement in these phenotypes. Overall, these baseline differences provide important clinical context for the observed stepwise increase in adverse outcomes across phenotypes.

Notably, patients in group D demonstrated the highest prevalence of prior heart failure among all geometry–function phenotypes. This finding may suggest that a substantial proportion of these patients had pre-existing chronic ventricular dysfunction and more advanced maladaptive remodeling before the index AMI event. In this context, prior studies have suggested that patients with *de novo* heart failure with reduced ejection fraction tend to exhibit a greater likelihood of left ventricular functional recovery and reverse remodeling compared with those with pre-existing heart failure (24). Conversely, a prior history of heart failure may reflect a lower probability of LVEF improvement and a more irreversible remodeling process, thereby contributing to worse long-term prognosis. Accordingly, the adverse prognosis observed in group D may partially reflect the coexistence of chronic pre-existing ventricular dysfunction in addition to acute post-infarction remodeling.

The differential prognostic impact of LV dilatation may be explained by distinct but interconnected remodeling pathways following myocardial injury (25). In the setting of preserved LVEF, ventricular enlargement likely represents an adaptive compensatory response to maintain stroke volume via the Frank–Starling mechanism (26). However, according to Laplace's Law, this balance is precarious as increased cavity radius inevitably elevates wall stress, stimulating further dilatation (25, 26). Conversely, when systolic function is already impaired, progressive dilatation reflects maladaptive remodeling driven by extensive myocyte loss—through necrosis and apoptosis—and chronic neurohormonal activation (26–28). These maladaptive processes are likely initiated by larger infarct burden and extensive myocardial injury. This process is further exacerbated by an imbalance of matrix metalloproteinases, leading to aberrant extracellular matrix degradation and increased chamber sphericity (26–28). These mechanisms create a vicious cycle where “dilatation begets more dilatation,” significantly accelerating clinical deterioration (25, 26).

Notably, a significant interaction by sex (*P* = 0.043) revealed a steeper risk gradient across these phenotypes in women. This heightened vulnerability likely stems from synergistic sex-specific mechanisms. First, estrogen deficiency, particularly post-menopause, activates the renin–angiotensin–aldosterone system and reduces nitric oxide levels, which directly accelerate collagen synthesis and increase diastolic LV stiffness (29). Second, women are more predisposed to coronary microvascular rarefaction and endothelial inflammation, promoting chronic myocardial ischemia and structural decay even without obstructive CAD (29–31). Third, at the molecular level, the female myocardium exhibits higher expression of proinflammatory genes and cytokines, which can exacerbate fiber-matrix deposition following injury (29–32). Finally, because female hearts have smaller baseline dimensions and higher elastance, any adverse geometric shift may confer a higher relative risk by reaching a threshold of structural collapse earlier than in men (29, 32). Although still speculative, these factors collectively suggest that adverse remodeling in women represents a more advanced state of biological failure. However, because LVEDD was not indexed to body surface area, the observed sex-related interaction should be interpreted cautiously. Some women classified as having non-dilated ventricles using the absolute LVEDD cutoff may have met criteria for LV dilatation after body size adjustment, potentially leading to reclassification from group C to group D. Therefore, the steeper risk gradient observed in women may reflect not only sex-specific biological vulnerability but also potential phenotype misclassification related to the use of an absolute LVEDD threshold.

This study may have important clinical implications for post-AMI risk stratification. While LVEF remains the cornerstone of clinical decision-making, our results suggest that reliance on systolic function alone may be insufficient to fully capture residual risk. The integration of LVEF with a simple geometric parameter such as LVEDD, available in routine echocardiographic evaluation, enables further stratification within both preserved and reduced LVEF categories. In particular, patients with concomitant ventricular dilatation and systolic dysfunction constitute a subgroup at markedly elevated risk who could benefit from closer surveillance, more intensive medical therapy, and potentially earlier consideration of advanced heart failure strategies. Conversely, mild ventricular dilatation in the setting of preserved systolic function should be interpreted with caution, as its prognostic significance may depend on the degree of structural remodeling. Our findings may support a more integrated and nuanced approach to assessment of ventricular remodeling beyond conventional LVEF-based risk stratification.

This study has several limitations. First, as an observational study based on a nationwide registry, residual confounding cannot be completely excluded despite comprehensive multivariable adjustment. In addition, information regarding sodium-glucose cotransporter-2 inhibitor use was not systematically available in the registry databases. Because these agents may influence ventricular remodeling and cardiovascular outcomes after AMI, the potential impact of these therapies could not be fully evaluated in the present analysis. Second, echocardiographic measurements, including LVEDD and LVEF, were obtained across multiple centers without centralized core laboratory adjudication, which may introduce interobserver variability. In addition, although LVEF was primarily assessed using the modified Simpson's biplane method, visual estimation was occasionally used when image quality was suboptimal. Furthermore, echocardiographic contrast agents were not routinely used across participating centers, which may have affected the accuracy and reproducibility of LVEF assessment in some patients. In addition, intracoronary thrombus was primarily identified using conventional coronary angiography without routine use of intracoronary imaging modalities, which may have limited the sensitivity and accuracy of thrombus detection in some patients. Third, LV geometry and systolic function were assessed at a single time point during index hospitalization, and thus dynamic changes in ventricular remodeling over time could not be captured. Fourth, the classification of LV geometry-function phenotypes was based on predefined cutoff values, which may oversimplify the continuous nature of ventricular remodeling, although our spline and sensitivity analyses partially addressed this limitation. In addition, LV chamber dimensions were not indexed to body surface area because indexed measurements were not consistently available in the registry databases. Therefore, some patients—particularly women or individuals with smaller body size—may have been misclassified regarding ventricular dilatation status when using an absolute LVEDD cutoff. This limitation may have partially influenced the neutral prognostic findings observed in patients with preserved LVEF and ventricular dilatation (group B). Therefore, although the integrated assessment of LV geometry and systolic function may provide practical prognostic information after AMI, these findings should be interpreted cautiously until validated in future prospective studies using indexed and sex-specific echocardiographic measurements.

In this nationwide cohort of patients with AMI, the integrated assessment of left ventricular geometry and systolic function provided clinically meaningful prognostic stratification beyond conventional assessment based solely on LVEF. Patients with concomitant LV dilatation and systolic dysfunction experienced a graded increase in long-term adverse outcomes, highlighting LV remodeling as a distinct high-risk phenotype. These findings underscore the clinical importance of incorporating simple echocardiographic indices of remodeling into post-infarction care to improve individualized risk assessment and optimize secondary prevention strategies.

## Data Availability

The data generated and/or analyzed during the current study are available from the corresponding authors upon reasonable request.

## Supplementary material

Supplementary Figure S1 Kaplan–Meier curves for other clinical outcomes according to LV geometry-function phenotypes. Blue line indicates group A (non-dilated/preserved), green line indicates group B (dilated/preserved), orange line indicates group C (non-dilated/reduced), and red line indicates group D (dilated/reduced). CVA, cerebrovascular accident; LV, left ventricular; NFMI, nonfatal myocardial infarction.
