# Supplementary material for: Integrated left ventricular geometry–function phenotypes and long-term outcomes after acute myocardial infarction
Source: Front Cardiovasc Med. 2026 Jun 22;13:1863946. doi: 10.3389/fcvm.2026.1863946 (PMC13333343; doi:10.3389/fcvm.2026.1863946)
Supplement: Supplementary file 1 [file Supplementaryfile1.docx]

**Supplementary Data S1.** Definition of left ventricular geometry–function phenotypes.

# Definition and Rationale

Left ventricular (LV) remodeling was characterized by combining geometric and functional indices derived from two-dimensional transthoracic echocardiography. Geometry was defined by the LV end-diastolic dimension (LVEDD, mm). Patients with LVEDD ≥ 53 mm were classified as dilated, whereas those with LVEDD < 53 mm were non-dilated. The threshold of 53 mm was selected in reference to prior literature identifying LVEDD values around 52–55 mm as markers of early ventricular dilatation or recovery of LV ejection fraction after intervention (1), and it lies within the upper-normal range proposed in echocardiographic chamber-quantification guidelines (2). Function was assessed by LV ejection fraction (LVEF, %). An LVEF ≤ 50 % was defined as reduced systolic function, while LVEF > 50 % was considered preserved, consistent with current European Society of Cardiology heart-failure classifications (3).

# Phenotypic Categorization

| Geometry (LVEDD) | Function (LVEF) | Phenotype | Group names |
| --- | --- | --- | --- |
| Non-dilated (< 53 mm) | Preserved (> 50 %) | Preserved geometry and function | Group A (reference) |
| Dilated (≥ 53 mm) | Preserved (> 50 %) | Impaired geometry but preserved function | Group B |
| Non-dilated (< 53 mm) | Reduced (≤ 50 %) | Preserved geometry but impaired function | Group C |
| Dilated (≥ 53 mm) | Reduced (≤ 50 %) | Impaired geometry and function | Group D |

# Analytic Implementation

These phenotypes were used to examine differences in baseline characteristics and clinical outcomes across progressive remodeling stages. The classification was performed in R (version 4.3.2) and verified by cross-checking with SPSS v25.0 (IBM Corp., Armonk, NY, USA).

# References

1. K. Ukita, Y. Egami, H. Nakamura, Y. Matsuhiro, K. Yasumoto, M. Tsuda, N. Okamoto, A. Tanaka, Y. Matsunaga-Lee, M. Yano, R. Shutta, Y. Sakata, M. Nishino and J. Tanouchi: Predictors of improvement of left ventricular systolic function after catheter ablation of persistent atrial fibrillation in patients with heart failure with reduced ejection fraction. *Heart Vessels*, 36(8), 1212-1218 (2021) doi:10.1007/s00380-021-01795-1

2. R. M. Lang, L. P. Badano, V. Mor-Avi, J. Afilalo, A. Armstrong, L. Ernande, F. A. Flachskampf, E. Foster, S. A. Goldstein, T. Kuznetsova, P. Lancellotti, D. Muraru, M. H. Picard, E. R. Rietzschel, L. Rudski, K. T. Spencer, W. Tsang and J. U. Voigt: Recommendations for cardiac chamber quantification by echocardiography in adults: an update from the American Society of Echocardiography and the European Association of Cardiovascular Imaging. *Eur Heart J Cardiovasc Imaging*, 16(3), 233-70 (2015) doi:10.1093/ehjci/jev014

3. T. A. McDonagh, M. Metra, M. Adamo, R. S. Gardner, A. Baumbach, M. Bohm, H. Burri, J. Butler, J. Celutkiene, O. Chioncel, J. G. F. Cleland, A. J. S. Coats, M. G. Crespo-Leiro, D. Farmakis, M. Gilard, S. Heymans, A. W. Hoes, T. Jaarsma, E. A. Jankowska, M. Lainscak, C. S. P. Lam, A. R. Lyon, J. J. V. McMurray, A. Mebazaa, R. Mindham, C. Muneretto, M. Francesco Piepoli, S. Price, G. M. C. Rosano, F. Ruschitzka, A. Kathrine Skibelund and E. S. C. S. D. Group: 2021 ESC Guidelines for the diagnosis and treatment of acute and chronic heart failure. *Eur Heart J*, 42(36), 3599-3726 (2021) doi:10.1093/eurheartj/ehab368
