# Supplementary material for: Integrated left ventricular geometry–function phenotypes and long-term outcomes after acute myocardial infarction
Source: Front Cardiovasc Med. 2026 Jun 22;13:1863946. doi: 10.3389/fcvm.2026.1863946 (PMC13333343; doi:10.3389/fcvm.2026.1863946)
Supplement: Supplementary file 2 [file Supplementaryfile2.docx]

**Supplementary Data S2.** Covariates for multivariable Cox models and rationale for variable selection

**Model Framework**

**Multivariable Cox proportional hazards models were fitted with sequential adjustment: Model 1 (unadjusted), Model 2 (age- and sex-adjusted), and Model 3 (fully adjusted).**

**Covariates in the Fully-Adjusted Model (Model 3).**

**• Demographics: age (continuous), sex (male sex versus female sex).**

**• Clinical status and comorbidities: body mass index, Killip functional classification at presentation (Killip II-III versus I), hypertension, diabetes mellitus, dyslipidemia, prior coronary artery disease (CAD), prior heart failure, prior cerebrovascular accident, smoking history, and estimated glomerular filtration rate.**

**• Angiographic and procedural factors: use of thrombolysis, multivessel CAD, left main coronary artery (LMCA) disease, access site (femoral access, non-femoral access), use of glycoprotein IIb/IIIa inhibitors, use of thrombus aspiration, use of intracoronary imaging, infarct-related artery (LMCA, left anterior descending coronary artery, left circumflex coronary artery, and right coronary artery), the American College of Cardiology/the American Heart Association (ACC/AHA) lesion characteristics (ACC/AHA B2 or C, ACC/AHA A or B1), and types of percutaneous coronary intervention (stenting, balloon angioplasty alone, and others).**

**• Discharge medications: aspirin, P2Y12 inhibitors, beta-blockers, renin-angiotensin-aldosterone system inhibitors, and statins.**

**Excluded Cardiac Size/Function Variables and Rationale.**

**• Left ventricular end-systolic diameter (LVESD): excluded from adjustment because of its close conceptual and mathematical relationship with left ventricular end-diastolic diameter (LVEDD) and left ventricular ejection fraction (LVEF), which jointly define the exposure phenotype (geometry × function). In other words, LVESD was summarized descriptively (Table 1) for clinical context but was not included as a covariate in multivariable models to avoid redundancy and over-adjustment.**

**• Other exposure-defining measures (LVEDD, LVEF): not additionally entered as covariates to avoid over-adjustment and construct redundancy.**
