# Supplementary material for: Integrated left ventricular geometry–function phenotypes and long-term outcomes after acute myocardial infarction
Source: Front Cardiovasc Med. 2026 Jun 22;13:1863946. doi: 10.3389/fcvm.2026.1863946 (PMC13333343; doi:10.3389/fcvm.2026.1863946)
Supplement: Supplementary file 3 [file Supplementaryfile3.docx]

**Supplementary Table S1**. Baseline characteristics of study participants according to left ventricular geometry–function phenotypes using alternative cutoffs (LVEF <40% and LVEDD ≥55 mm).

|  | Group A | Group B | Group C | Group D | *P*-value |
| --- | --- | --- | --- | --- | --- |
|  | **(n=15,238)** | **(n=2,253)** | **(n=1,245)** | **(n=928)** | **for trend** |
| Age, years | 62.71±12.31 | 62.17±12.28 | 67.46±12.67 | 66.18±12.64 | <0.001 |
| Age ≥75 years | 3046 (20.0) | 421 (18.7) | 434 (34.9) | 277 (29.8) | <0.001 |
| Male sex | 11591 (76.1) | 1967 (87.3) | 820 (65.9) | 740 (79.7) | 0.398 |
| Use of EMS | 2551 (16.7) | 350 (15.5) | 224 (18.0) | 136 (14.7) | 0.314 |
| BMI, kg/m^2^ | 24.26±3.23 | 25.36±3.65 | 23.20±3.41 | 23.81±3.32 | <0.001 |
| Killip class II-III | 1686 (11.2) | 334 (15.1) | 328 (26.5) | 357 (39.3) | <0.001 |
| Comorbidities |  |  |  |  |  |
| Hypertension | 7198 (47.2) | 1164 (51.7) | 615 (49.4) | 507 (54.6) | <0.001 |
| Diabetes mellitus | 3750 (24.6) | 563 (25.0) | 398 (32.0) | 368 (39.7) | <0.001 |
| Dyslipidemia | 1977 (13.0) | 258 (11.4) | 120 (9.6) | 108 (11.6) | 0.001 |
| Prior CAD | 1080 (7.1) | 185 (8.2) | 95 (7.6) | 97 (10.4) | <0.001 |
| Prior heart failure | 89 (0.6) | 27 (1.2) | 21 (1.7) | 44 (4.8) | <0.001 |
| Prior CVA | 853 (5.6) | 108 (4.8) | 118 (9.5) | 87 (9.4) | <0.001 |
| Smoking history | 8660 (58.3) | 1472 (66.9) | 600 (49.8) | 517 (57.5) | 0.199 |
| Family history of CAD | 1183 (8.0) | 149 (6.8) | 66 (5.4) | 66 (7.3) | 0.007 |
| Use of thrombolysis | 109 (0.7) | 38 (1.7) | 6 (0.5) | 6 (0.6) | 0.480 |
| Multivessel CAD | 7483 (49.1) | 1184 (52.6) | 722 (58.0) | 652 (70.3) | <0.001 |
| LMCA disease | 585 (3.8) | 85 (3.8) | 69 (5.5) | 66 (7.1) | <0.001 |
| Femoral approach | 7479 (49.1) | 938 (41.6) | 759 (61.0) | 483 (52.0) | <0.001 |
| Use of GPIIb/IIIa inhibitors | 1626 (10.7) | 378 (16.8) | 134 (10.8) | 84 (9.0) | 0.183 |
| Use of thrombus aspiration | 2786 (18.3) | 362 (16.1) | 298 (23.9) | 116 (12.5) | 0.186 |
| Use of intracoronary imaging | 3719 (24.4) | 763 (33.9) | 252 (20.2) | 206 (22.2) | 0.903 |
| Infarct-related artery |  |  |  |  | <0.001 |
| LMCA | 267 (1.7) | 53 (2.3) | 37 (3.0) | 42 (4.5) |  |
| LAD | 7023 (46.1) | 984 (43.7) | 875 (70.3) | 538 (58.0) |  |
| LCX | 2843 (18.7) | 490 (21.8) | 108 (8.7) | 123 (13.2) |  |
| RCA | 5105 (33.5) | 726 (32.2) | 225 (18.1) | 225 (24.3) |  |
| ACC/AHA lesion type B2/C | 12497 (85.0) | 1915 (87.5) | 1052 (88.0) | 785 (87.6) | <0.001 |
| PCI strategies |  |  |  |  | 0.617 |
| Stenting | 14278 (94.7) | 2137 (95.6) | 1177 (95.4) | 864 (94.2) |  |
| Balloon angioplasty alone | 694 (4.6) | 84 (3.8) | 47 (3.8) | 45 (4.9) |  |
| Others | 100 (0.7) | 14 (0.6) | 10 (0.8) | 8 (0.9) |  |
| eGFR, mL/min/1.73m^2^ | 83.23±22.53 | 80.29±25.36 | 72.60±25.46 | 66.74±29.09 | <0.001 |
| Echocardiographic profiles |  |  |  |  |  |
| LVEF, % | 55.82±8.12 | 51.75±7.61 | 35.10±4.78 | 32.05±6.32 | <0.001 |
| LVESD, mm | 31.55±6.50 | 40.12±7.58 | 36.15±9.08 | 48.13±10.56 | <0.001 |
| LVEDD, mm | 47.95±5.25 | 58.31±17.67 | 48.64±7.02 | 60.72±8.49 | <0.001 |
| STEMI as a final diagnosis | 7276 (47.8) | 1084 (48.1) | 810 (65.1) | 424 (45.7) | <0.001 |
| Medications |  |  |  |  |  |
| Aspirin | 15212 (99.8) | 2250 (99.9) | 1244 (99.9) | 927 (99.9) | 0.387 |
| P2Y12 inhibitors | 15201 (99.8) | 2248 (99.8) | 1241 (99.7) | 924 (99.6) | 0.302 |
| Beta-blockers | 12818 (84.1) | 1827 (81.1) | 1014 (81.5) | 755 (81.4) | <0.001 |
| RAAS inhibitors | 14257 (93.6) | 2126 (94.4) | 1135 (91.2) | 877 (94.5) | 0.607 |
| Statins | 14739 (96.7) | 2179 (96.7) | 1181 (94.9) | 871 (93.9) | <0.001 |

Continuous variables are presented as mean ± standard deviation, and categorical variables as numbers with percentages. Group definitions were based on combined geometric and functional classification using alternative thresholds to assess the robustness of the primary analysis. ACC/AHA, American College of Cardiology/American Heart Association; BMI, body mass index; CAD, coronary artery disease; CVA, cerebrovascular accident; eGFR, estimated glomerular filtration rate; EMS, emergency medical service; GPIIb/IIIa, glycoprotein IIb/IIIa; LAD, left anterior descending coronary artery; LCX, left circumflex coronary artery; LMCA, left main coronary artery; LVEDD, left ventricular end-diastolic diameter; LVEF, left ventricular ejection fraction; LVESD, left ventricular end-systolic diameter; PCI, percutaneous coronary intervention; RAAS, renin-angiotensin-aldosterone system; RCA, right coronary artery; STEMI, ST-segment elevation myocardial infarction.
