# Supplementary material for: Integrated left ventricular geometry–function phenotypes and long-term outcomes after acute myocardial infarction
Source: Front Cardiovasc Med. 2026 Jun 22;13:1863946. doi: 10.3389/fcvm.2026.1863946 (PMC13333343; doi:10.3389/fcvm.2026.1863946)
Supplement: Supplementary file 4 [file Supplementaryfile4.docx]

**Supplementary Table S2**. HRs and 95% CI for clinical outcomes according to left ventricular geometry–function phenotypes using alternative cutoffs (LVEF <40% and LVEDD ≥55 mm).

|  |  |  | HR with 95% CI | | |
| --- | --- | --- | --- | --- | --- |
|  | **Study group** | **Events** | **Unadjusted model** | **Age-sex adjusted model** | **Fully-adjusted model** |
| MACCE | Group A | 2003 (13.4) | 1 (reference) | 1 (reference) | 1 (reference) |
|  | Group B | 359 (16.3) | 1.23 (1.10-1.37) | 1.27 (1.13-1.42) | 1.24 (1.09-1.40) |
|  | Group C | 316 (26.5) | 2.22 (1.97-2.50) | 1.90 (1.69-2.14) | 1.57 (1.37-1.80) |
|  | Group D | 292 (33.1) | 2.90 (2.56-3.27) | 2.64 (2.33-2.98) | 1.92 (1.66-2.21) |
| All-cause death | Group A | 588 (3.9) | 1 (reference) | 1 (reference) | 1 (reference) |
|  | Group B | 115 (5.2) | 1.32 (1.08-1.62) | 1.36 (1.11-1.66) | 1.25 (0.99-1.57) |
|  | Group C | 134 (11.2) | 3.06 (2.54-3.69) | 2.21 (1.83-2.67) | 1.65 (1.33-2.05) |
|  | Group D | 132 (14.9) | 4.13 (3.42-4.99) | 3.24 (2.68-3.92) | 2.21 (1.77-2.76) |
| Cardiac death | Group A | 267 (1.8) | 1 (reference) | 1 (reference) | 1 (reference) |
|  | Group B | 51 (2.3) | 1.29 (0.96-1.75) | 1.33 (0.98-1.79) | 1.14 (0.79-1.64) |
|  | Group C | 79 (6.6) | 3.95 (3.07-5.08) | 2.87 (2.23-3.70) | 2.04 (1.52-2.74) |
|  | Group D | 75 (8.5) | 5.14 (3.98-6.64) | 4.05 (3.13-5.25) | 2.75 (2.03-3.72) |
| Non-cardiac death | Group A | 321 (2.1) | 1 (reference) | 1 (reference) | 1 (reference) |
|  | Group B | 64 (2.9) | 1.35 (1.03-1.76) | 1.38 (1.06-1.81) | 1.32 (0.97-1.79) |
|  | Group C | 55 (4.6) | 2.31 (1.74-3.08) | 1.66 (1.25-2.21) | 1.30 (0.93-1.80) |
|  | Group D | 57 (6.5) | 3.28 (2.47-4.35) | 2.56 (1.93-3.40) | 1.73 (1.24-2.41) |
| NFMI | Group A | 330 (2.2) | 1 (reference) | 1 (reference) | 1 (reference) |
|  | Group B | 60 (2.7) | 1.23 (0.94-1.62) | 1.27 (0.96-1.67) | 1.10 (0.80-1.51) |
|  | Group C | 42 (3.5) | 1.72 (1.25-2.37) | 1.56 (1.13-2.15) | 1.15 (0.79-1.68) |
|  | Group D | 36 (4.1) | 2.03 (1.44-2.87) | 1.92 (1.36-2.72) | 1.31 (0.89-1.94) |
| Any unplanned revascularization | Group A | 1020 (6.8) | 1 (reference) | 1 (reference) | 1 (reference) |
|  | Group B | 168 (7.6) | 1.12 (0.95-1.32) | 1.12 (0.95-1.32) | 1.09 (0.91-1.30) |
|  | Group C | 98 (8.2) | 1.30 (1.06-1.60) | 1.30 (1.05-1.60) | 1.17 (0.93-1.48) |
|  | Group D | 77 (8.7) | 1.41 (1.12-1.78) | 1.41 (1.12-1.77) | 1.12 (0.87-1.45) |
| CVA | Group A | 241 (1.6) | 1 (reference) | 1 (reference) | 1 (reference) |
|  | Group B | 42 (1.9) | 1.18 (0.85-1.63) | 1.25 (0.90-1.74) | 1.25 (0.87-1.81) |
|  | Group C | 33 (2.8) | 1.85 (1.29-2.66) | 1.56 (1.08-2.25) | 1.43 (0.96-2.14) |
|  | Group D | 27 (3.1) | 2.07 (1.39-3.09) | 1.90 (1.28-2.84) | 1.41 (0.88-2.26) |
| Cardiovascular readmission | Group A | 312 (2.1) | 1 (reference) | 1 (reference) | 1 (reference) |
|  | Group B | 80 (3.6) | 1.75 (1.37-2.24) | 1.95 (1.52-2.49) | 1.84 (1.40-2.43) |
|  | Group C | 93 (7.8) | 4.08 (3.24-5.14) | 3.11 (2.46-3.93) | 2.28 (1.75-2.99) |
|  | Group D | 117 (13.2) | 7.16 (5.79-8.86) | 6.32 (5.10-7.83) | 4.42 (3.43-5.70) |

HRs were estimated using Cox proportional hazards models with sequential adjustment: unadjusted, age- and sex-adjusted, and fully adjusted models. Covariates included in the fully adjusted model are described in **Supplementary Data S2**. Group A (non-dilated with preserved function) was used as the reference category.
