# Supplementary figures and images for: Integrated left ventricular geometry–function phenotypes and long-term outcomes after acute myocardial infarction
Source: Front Cardiovasc Med. 2026 Jun 22;13:1863946. doi: 10.3389/fcvm.2026.1863946 (PMC13333343; doi:10.3389/fcvm.2026.1863946)

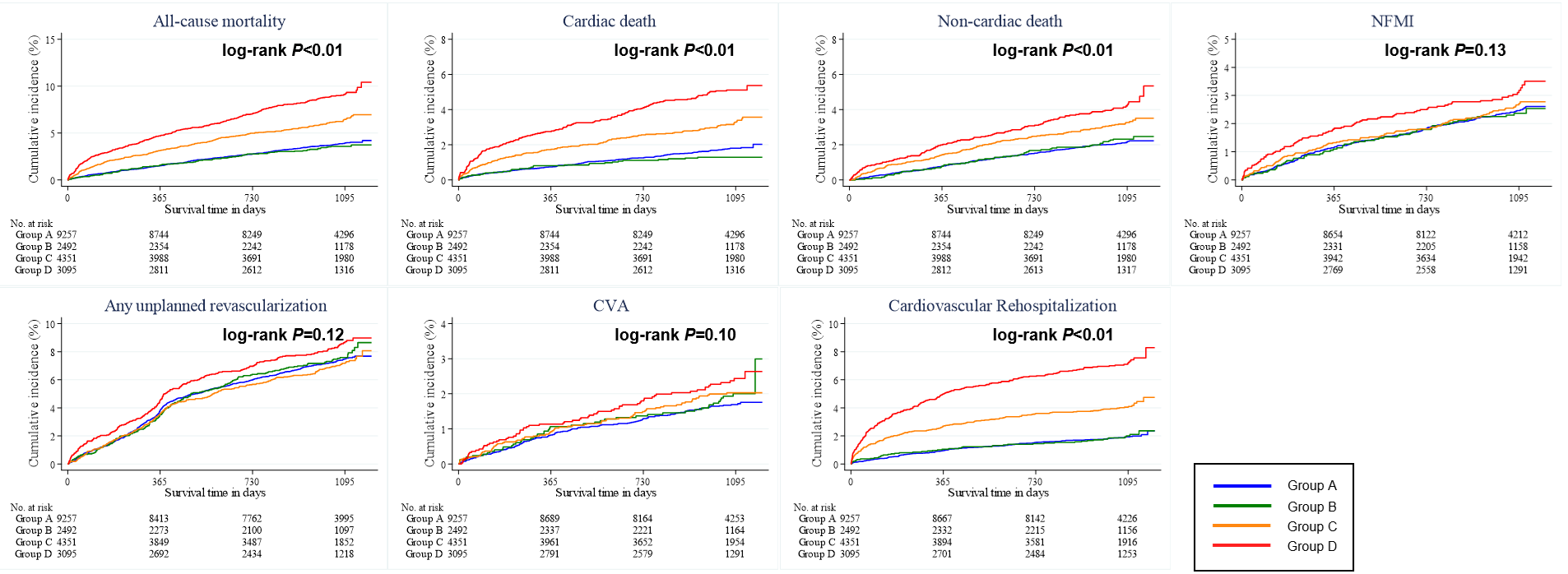

Supplement: Supplementary file 5 [file Image1.tif]
